# Patterns of communicating care and caring in the intensive care unit

**DOI:** 10.1002/nop2.1061

**Published:** 2021-09-18

**Authors:** Hanan Subhi Al‐Shamaly

**Keywords:** caring, communication enablers/facilitators/barriers/obstacles/challenges, documentation, focused ethnography, intensive care nurses, technology, touch

## Abstract

**Aim:**

To explore the perceptions and experiences of nurses in communicating the care and caring in the intensive care unit (ICU).

**Design:**

A focused ethnography.

**Methods:**

This study was conducted in an Australian metropolitan hospital, in which data were gathered from multiple sources: participant observations, document reviews, interviews, and participant's additional written information ‐ over six months (April‐September, 2014). The data were analysed thematically.

**Findings:**

This study addressed inclusively communicating care and caring to patients, families, nurses and other health professionals in ICU. The findings identified main themes concerning the changing patterns of communicating the care and caring in ICU, various patterns of communication used, enablers and barriers of communicating care and caring, and significant issues in communicating care and caring in ICU. Documentation of patients’ psychological and emotional needs, and nurses’ caring behaviours are crucial. These findings need further consideration from all stakeholders.

## 1 INTRODUCTION

ICU is considered a high‐pressure working environment due to the complex nature of the work. Caring in the context of ICUs combines humanistic approaches to caring with heavy reliance on the most advanced technology to provide high‐quality care to critically ill patients (Limbu et al., 2019; Marik, 2010). The potential for technological dehumanization is a challenge because patient care requires substantial use of technologies that can override other factors of care and create feelings of detachment (Lopes de Souza et al., 2019; McGrath, 2008). Unconscious or mechanically ventilated (MV) patients experience communication difficulties (Anna et al., 2021; Karlsson et al., 2012), which makes the provision of care different from that given to other patients; therefore, it is vital to maintain communicating the care and caring so that patients can be understood and humanized (Karlsson et al., 2012; Nin Vaeza et al., 2020). Nursing care is a part of the broad concept of caring, as caring concept is complex and nebulous. Communication in ICU is not only limited to patients but also extends to families and health team members where intra/inter/transpersonal communication is important (Mahvar et al., 2020; Saldaña et al., 2015). Therefore, communicating care and caring could include the care that person needs (e.g. psychological support for families) or nature of communication (e.g. caring mannerism for died patients). In this paper, the communication of care and caring can include from nurses to patients/families/nurses/other health team members, and vice versa.

## 2 BACKGROUND

As a concept, caring is inextricably intertwined with nursing. There is a plethora of literature devoted to the concept of caring, but it is nebulous and complex, and it is never clearly defined. Numerous theoretical and operational perspectives of caring in the context of nursing have emerged over time. There is the ongoing dialogue and debate about what constitutes caring in the ever‐expanding domains of nursing practice. The association of care with other words in current nursing language has meaning when it is used in compound nouns. For example, nurses use the terms “care giving” (Wolf, 1986), “care plans” (Kolcaba, 1995), “nursing care” (Tulek et al., 2018), “plan of care” (Lea & Watson, 1996), “duty of care” (Kelly, 2010), “health care” (Chao, 1992), “basic, fundamental or essential care” (Crisp et al., 2012) and “intensive care” (Hogg, 1994). In the literature, the words “nursing,” “nursing practice” and “nursing care” can be readily interchanged (Kozier et al., 1989). Leininger (1991, p. 4) defined caring as “those actions and activities directed toward assisting, supporting, or enabling another individual or group with evident or anticipated needs to ameliorate or improve a human condition or lifeway.” Wikberg and Eriksson (2008) purport that it is the subject of nursing science, while nursing itself comprises what nurses do. Eriksson (1997) further discusses nursing and caring relations when describing the three different perspectives of nursing: first, caring as the innermost core of nursing; second, nursing based on the nursing process; and third, the structure of the nursing care plan. All three perspectives are key to good nursing care, although nursing does not necessarily involve caring (Eriksson, 1997). Two caring aspects are consistently identified: the instrumental/technical aspects and the expressive/affective/psychological aspects (Arthur et al., 1999; Bégat & Severinsson, 2001; Watson & Lea, 1997). Kuhse (1997) pointed out that care has two connotations: first, it is an “emotional response” such as worry and inclination, for example Johnstone’s (1994) description of caring as a feeling parallel to sympathy, empathy and compassion; and second, it is “providing for”: doing something for another person, for example Griffin (1983) description of caring as seeing to somebody's needs. Bourgeois (2006) identified that an “archive of caring” exists for nursing as “caring as knowing,” “caring as being” and “caring as doing.” This lack of understanding, definition or agreed theoretical perspective is a common theme in the caring literature and underpins the debate about the centrality of caring in the nursing paradigm. Caring in nursing remains contentious, making it difficult to reach a consensus about the definitions, perspectives, components and process of caring (Paley, 2001; Sebrant & Jong, 2021; Smith, 1999) Therefore, the researcher considered “nursing care” as a part of the broad “caring” concept.

The nurses in ICU focus on patients and their families to prevent deterioration and improve the patient's condition (Sole et al., 2009), and to deal with life‐saving interventions during acute physiological crises, with a particular focus on medical needs and access to technology (Marik, 2010). Good communication is vital to the assessment of symptoms and in promoting patients’ participation in decision‐making about treatment plans and end‐of‐life (EOL) decisions (Happ et al., 2004) and giving care to families at this stage (Almansour & Abdel Razeq, 2021; Rivera‐Romero et al., 2019). There are various factors that affect communicating care and caring in ICU (Ganz, 2019; Happ et al., 2011). There are different means and measures to assist patients in communicating (Shiber et al., 2016). With some patients, especially MV patients, communication can be limited to verbal responses of yes/no or non‐verbal answers, such as eye blinking and touching (Karlsson, Forsberg, et al., 2012; ten Hoorn et al., 2016). Radtke et al. (2012) found that nurses’ evaluations of communication can vary. Despite the benefits of taking time for caring communication, some nurses prioritize medical treatment over communication, while others consider assistive communication methods to be time‐consuming and impractical, emotionally exhausting and inappropriate for ICU patients. This may lead ICU nurses to use less verbal communication when caring for unconscious patients than they would with verbally responsive patients, and to communicate less with unconscious patients than with conscious patients (Alasad & Ahmad, 2005; Happ et al., 2014). Karlsson, Forsberg, et al. (2012) emphasized that for nurses to communicate caring with patients, they need to set aside time to build a trusting relationship; to read, interpret and listen to the patient's way of communication; and to be aware of their own attitude, body language, words, tune and when they touch the patient.

Furthermore, ICU nurses need to deal with patients and their families, and other healthcare colleagues from different disciplines (Communicating with Patients & Colleagues, 2021; Karlsson, Forsberg, et al., 2012). Accordingly, caring communication also includes patients’ families and other health professionals, which raises the question of what constitutes caring communication in ICU. The aim of this study was to explore the perceptions and experiences of nurses in communicating the care and caring in ICU.

## 3 THE STUDY

### 3.1 Design

Focused ethnography (FE) was chosen to examine nurses’ beliefs, behaviours, social interactions and practices (Magilvy et al., 1987; Roberts, 2009) about communicating care and caring in ICU. The researcher employed FE because of its congruence with the research question, which centres on describing experiences in a cultural context (Higginbottom et al., 2013; Richards & Morse, 2013).

#### 3.1.1 Focused ethnography

The term “focused ethnography” consists of two words: focused and ethnography. Ethnography was defined differently by scholars (De Chesnay, 2015; Fetterman, 1998; Holloway & Wheeler, 2010; Leininger, 1985; Schneider et al., 2007; Willis & Anderson, 2010). Polit and Beck (2017), Roper and Shapira (2000), Spradley (1980) and Oliffe (2005) all highlight that ethnography is learning *from* people and is distinct from studying or learning *about* people. Harris and Johnson (2006, p. 5) defined ethnography as “a written description of a particular culture ‐ the customs, beliefs, and behaviour ‐ based on information collected through fieldwork.” Goodson and Vassar (2011, p. 2) described ethnography as “a social research method occurring in natural settings characterized by learning the culture of the group under study and experiencing their way of life before attempting to derive explanations of their attitudes or behaviour.” Muecke (1994, pp. 189–190) provides a succinct yet comprehensive description of ethnography as:

A written description of a people that focuses on selected aspects of how they lead their routine, remarkable and ritual lives with each other in their environment and of the beliefs and customs that comprise their common sense about their world.

Knoblauch (2005) used the term “focused” because FE concentrates on small elements of a culture. Muecke (1994) used the term FE to describe time‐limited exploratory studies in a fairly discrete community or organization with a limited number of key informants having knowledge of problem or phenomenon of study. For the purpose of this study, Muecke’s (1994) definition of FE was adopted as this exploratory study focused on a particular culture or phenomenon—communicating caring in an ICU—in a limited time frame and with a limited number of participants.

The study was reported according to Consolidated Criteria for Reporting Qualitative Research (COREQ) Checklist (File S1). This study was undertaken by Ms. H.A, a Registered Nurse (RN) with several years of experience in intensive care, perioperative and emergency nursing. Research Ethics Committee approval from the human research ethics committees at both the university and the hospital was obtained prior to commencement of the study. The study was undertaken in ICU of one of the largest metropolitan private hospitals in Queensland, Australia.

### 3.2 Setting

The study was conducted at the adult ICU of a large private hospital in the Queensland metropolitan area, Australia. There is a low nurse‐to‐patient ratio (1:1 or 1:2) in this ICU (Almerud et al., 2007; Kim et al., 2012; Marik, 2010), where nurses are continuously at the bedside and monitor all aspects of the patients’ health status.

The physical layout of the ICU starts from the entrance site. There is a volunteer clerk outside the unit, who cooperates with the ICU staff and receptionist to arrange relatives’ visiting times. Next to the entrance door, there is a waiting room for visitors with facilities and self‐service refreshments and utilities. On the wall, there are brochures, leaflets and other educational materials for visitors.

This ICU consists of 19 beds (17 in open rooms and 2 in isolated rooms), divided into two wings. The right wing includes bed numbers 1–13. There are six beds in the left wing: beds 14–19, which includes the isolation rooms 18 and 19. Beds 1–6 are allocated to conscious patients because they are away from the nurses’ station and experience the least noise from staff activities, especially at night. There are a number of offices in the left wing for the medical staff, as well as a rest room for the Medical Officer (MO) on‐call. Patients’ rooms have electrical bed and a locker. Each room has oxygen and suction units and a ceiling‐mounted computer for nurses’ usage. There is also a TV on the wall of each patient's room, and each room can be closed by disposable blue curtains. In front of each bed, there is a desk and chair with space for the nurses to sit and write their records and notes.

There is the staff's tearoom at the end of the right wing, which faces the respiratory room with an arterial blood gas (ABG) machine. There are two nurses’ stations: the main one is the largest and faces the Clinical Nurse Manager's (CNM) office; and the small one is located in front of the first six beds of the right wing. The main nurses’ station includes the in‐charge nurse, receptionist, and the doctors’ desks and five computers for doctors and nurses to find any information, procedure or results. In the nurses’ station, there are an X‐ray machine (where the X‐ray technician can obtain X‐ray films) and X‐ray screens for doctors to view these films. There is a white board where the patients’ names are allocated to their room number along with their specialist, the intensivist and MO who are on‐duty/call for the day. There are two mobile telephones with security video calls. The receptionist (or present staff) responds to the entry bell by pressing a button on the telephone to open the door for the caller. In addition, there is a landline telephone only used for emergency calls. The main nurses’ station also holds cabinets with drawers of numerous folders and forms for doctors’, health professionals’ and nurses’ requirements. On the shelves, there are many “thank you” letters to staff, and the staff communication book is located in the in‐charge nurse's space. From the nurses’ station, there are exits to the utility room where basins, urinals and bedpans are stored; to the medication's rooms; and to additional storeroom for other equipment and tools. The middle of the unit has another clean utility room and bathrooms. The conference/family meeting room is at the exit of the unit.

The hub of the unit is the main nurses’ station, where all staff movements originate and return on a regular basis. The station is rarely vacant, and on several occasions, as many as 30 staff could be present and engaged in different group interactions simultaneously. Nursing staff begin their shift by entering the station and checking the allocation book to find out who their patients are for the day. They then accept handover from the nurse of the previous shift.

### 3.3 Sample

The inclusion criteria for this study were as follows: RN, either male or female; employed full‐time worker; working for at least one year in the unit; working alternative shifts; and willing to be interviewed and observed in the practice setting. A purposive sample of 38 RNs consented to participate in this study. Three participants withdrew for personal reasons. Subsequently, 35 was the total number of the participants. Table 1 provides the demographics of the participants.

**TABLE 1:** Basic demographic data sheet for study participants

Age	Gender	Marital status	Ethnic background	Languages	Religion	Education	Years’ experience in ICU
22–60	F: 29	Married 25	Australian 25	English	Catholic 12	Master's 6	Range 1–34
	M: 6	Partnered 1	New Zealander 1	Indian	Protestant 3	Bachelor's 17	1 y = 1
		Engaged 1	British 4	Mandarin	Anglican 1	Diploma 2	3 y = 1
		Divorced 1	Irish 1	Chinese	Church of England 1	Postgraduate certificate 7	4 y = 1
		De facto 3	Indian 1	Tagalog	Buddhist 2	Graduate certificate for critical care 3	5 y = 1
		Single 4	Filipino 1	Malayalam	Hindu 2		6 y = 4
			Thai 1		Pentecostal 1		7 y = 1
			Chinese 1		Honours and respects all religions 1		8 y = 4
					No religious affiliation 12		9 y = 1
							10 y = 3
							12 y = 2
							13 y = 1
							14 y = 1
							15 y = 3
							16 y = 1
							19 y = 1
							22 y = 1
							25 y = 1
							28 y = 1
							30 y = 2
							32 y = 1
							34 y = 3
Total participants	35						

### 3.4 Data collection

The recruitment process began with meeting the CNM, who then introduced the researcher to the staff. The researcher provided the CNM with letters of invitation, which she in turn distributed to nursing staff and placed flyers around the nurses’ stations and the staff tearoom. The researcher then personally contacted those who responded and provided three documents: information sheet, informed consent form and demographic questionnaire. The researcher is a Registered Nurse with long experience in different fields of nursing (e.g. intensive care, emergency and perioperative). The researcher has no previous relationship with participants.

All participants provided their written consent to participate prior to data collection. Participant are identified by “P” followed by a number (e.g. P1) for anonymity. Data were also gathered from participant observations, document reviews, interviews, and further written information from participants. Data were gathered in short and long rotating shifts; morning, evening, and night; weekdays and weekends; and public holidays.

#### 3.4.1 Participant observation

Participant observation is considered the fundamental ethnographic research method (Fife, 2005). Observation without participation can be used to gain a rich description of the setting, activities and the participants to describe and explain their actions in context (Hennink et al., 2011). This assists in both understanding the important issues in the designated setting (Boswell & Cannon, 2011) and interpreting the experiences of the studied group (Holloway & Wheeler, 2010). The researcher started the observation period by exploring the physical and social structures of the unit and experiencing the everyday running and routines of ICU, as Holloway and Wheeler (2010) recommended. Observation assisted the researcher to contextualize the attitudes, values and emotions of the participants. An unstructured observation method was used to obtain detailed descriptions of participants’ behaviours either as they occurred or shortly afterwards by compiling field notes or completing the researcher's reflective journal. At times, the participants were observed for more than two shifts because they were interacting with other participants and events. Patients, families and other personnel who were not the focus of this study were informed of the reason for the presence of an observer researcher. Participant observation was conducted with 1,632 hr.

#### 3.4.2 Documentation review

Reviewing documents such as nurses’ records, policies and procedures allowed the researcher to access data that were difficult to acquire by direct observation and interviewing (Holloway & Wheeler, 2010). Document reviews occurred concurrently with the participant observation period; as the researcher took field notes, she also examined documents as the study progressed. The researcher read the narrative nurses’ notes, which contained patient observations, progress of the patient's condition, statements to specify the nursing care pertinent to patients and their families and their response to this care. Further clarifications were obtained by interviews as regards the participants’ notes.

#### 3.4.3 Interviews

Interviews allow the exploration of unique cases and unexpected responses that might have given profound insights into the phenomenon being studied (Taylor et al., 2006). Ethnographers depend on interviews to understand the participants’ personal worlds (Wolf, 2012). In interviews, the researcher is mentally projected into the ethnographic experiences described by the participants (Bauman & Adair, 1992). Moreover, interviews may be the only method available to collect certain data that are difficult to obtain through participant observation (Hammersley & Atkinson, 2007; Polit & Beck, 2017). Therefore, the use of interviews was considered by this researcher to be essential and complementary to the other forms of data collection for this study.

Participants were interviewed after they were observed in this study. The researcher used the Nurses’ Interview Guide (Bryman, 2012; Roberts, 2020), which included asking participants’ permission for a digital audio recording of the interview. The interviews were arranged to suit the participants’ schedules in days. Interviews were conducted at workplace or out‐of‐work hours (e.g. family conference room, participants’ home). The researcher conducted pilot face‐to‐face, semi‐structured interviews with four participants, which enabled pre‐testing and improvement of the interview guide and process (Bryman, 2012). Each interview started with broad, general and open question such as “How do you communicate that you care to your conscious/unconscious patients?” The questions were narrowed from broad to specific questions, probes and prompts as “Tell me more about that.” Such prompts were used to clarify content and augment the information provided. The researcher avoided indicating responses in these questions, posed one question at a time. The researcher took notes before, during and after the interviews. The average interview lasted 1 to 1.5 hr. There were 44 follow‐up interviews to obtain further clarifications about observational periods with total of formal interviews (*N* = 79) and informal conversations (*N* = 16). The number of participant interviews is variant. Some participants had only one interview, others had 2–3 interviews, and the only one had 4 interviews (CNM), as she plays different roles (manager, in‐charge and assisting as allocated nurse).

#### 3.4.4 Participant's additional written information forms

Twenty‐six Participant's additional written information forms (PAWIFs) were filled by participants for further information, which allows participants to feel comfortable about self‐disclosure in private and at a convenient time (Smith‐Sullivan, 2008). Twenty‐six PAWIFs were collected, and the length of PAWIFs varied from a single paragraph to seven pages. This focused ethnography is recognized by excessive data saturation due to the lengthy time, and triangulation of data methods was used (Fusch & Ness, 2015). Data saturation is reached when there is enough information to replicate the study (O’reilly & Parker, 2013; Walker, 2012), when the ability to obtain additional new information has been attained (Guest et al., 2012) and when further coding is no longer feasible (Guest et al., 2012), which explicates the level to which new data repeat what was expressed in previous data (data replication) (Fusch & Ness, 2015), and after six months of the fieldwork, it became apparent that no new data were forthcoming (Hennink et al., 2017, 2019). Therefore, the researcher determined that the study had reached data saturation (Polit & Beck, 2017).

### 3.5 Data analysis

Data were inductively and thematically analysed by the researcher. There is no specific protocol for analysis in ethnographic research; therefore, the researcher reviewed a number of approaches (Braun & Clarke, 2006; Chuang & Abbey, 2009; Padgett, 2012; Polit & Beck, 2017; Whitehead, 2013). From this emerged a modified six‐phase analysis process. The data from field notes, reflective journal, documentation, interviews and PAWIFs were segmented, compared, contrasted, synthesized, categorized and conceptualized to identify common codes, categories/subthemes and core themes, from which a mental map of the findings was constructed and reconstructed to capture the core concepts in the data set (Hennink et al., 2011). NVivo 11^®^ data management software was used to facilitate the analysis.

The researcher did not return the interview transcripts to participants for comments, as she conducted instant member checking through various “good interviewing” strategies as seeking clarification by probing, paraphrasing, using open‐ended questions and listening with an interpretive intent (McConnell‐Henry et al., 2011).

Rigour of the study was ensured through several means. Credibility was ensured by the prolonged engagement of the researcher in the field and triangulation of the data methods, which provided converging conclusions to provide rich and vivid descriptions; dependability through the consistency, triangulation and trail audit in the methods of data collection and analysis; confirmability, an audit trail was created where all steps taken in the research process are outlined and made available in this document for scrutiny; transferability, by clearly outlining the context of the study and the rationale for its undertaking such as establishing the participant inclusion criteria and articulating the analysed data; and reflexivity through the use of researcher's reflective journaling about preconceived biases, preferences and preconceptions that may have influenced a situation or interpretation of data (Polit & Beck, 2017).

### 3.6 Trustworthiness

To ensure the rigour of the study, the researcher employed credibility through the prolonged engagement of the researcher in the field, and data methods triangulation provided converging conclusions to provide rich and vivid descriptions; dependability through the consistency, triangulation and trail audit in the methods of data collection and analysis; and confirmability by the researcher's checking of the codes and analysis with expert researchers to obtain data accuracy, relevance or meaning. To ensure transferability, the researcher clearly outlined the context of the study and the rationale for its undertaking such as establishing the participant inclusion criteria and articulating the analysed data. Reflexivity was achieved through the use of reflective journaling about preconceived biases, preferences and preconceptions that the researcher may have to influence a situation or interpretation of data (Polit & Beck, 2017).

### 3.7 Ethical considerations

Research Ethics Committee approval has been granted by ethics committees at both the university and the hospital. Informed consent was obtained from each participant before data were collected. The researcher provided the participants with a research information sheet that included the objectives of the study, methods of collecting information, risk level, confidentiality guarantees and anonymity. Participants were informed that they could withdraw at any time without prejudice. Participants have been de‐identified and coded with the letter P and number (e.g. P1) to ensure anonymity. The researcher kept participants’ information on electronic computer files, which were password‐protected and regularly backed up to reduce the risk of damage or data loss. Patients and other staff who were not at the centre of this study were informed of the reason for the researcher's presence, and their permission was taken.

## 4 FINDINGS

**FIGURE 1:**
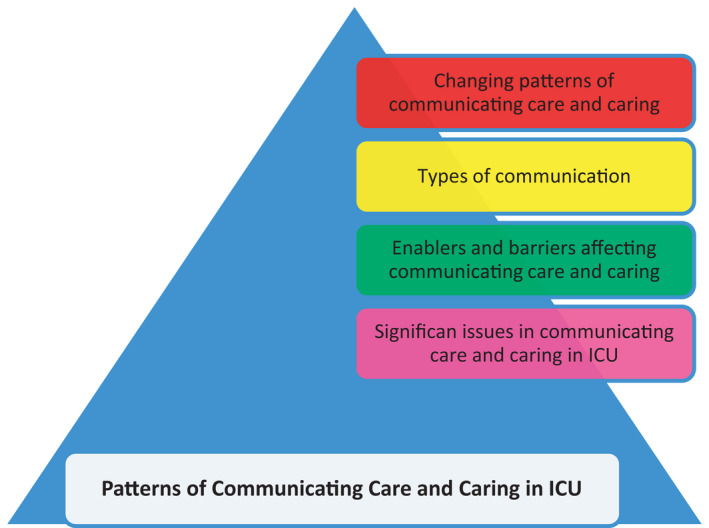
Patterns of communicating care and caring in ICU

The data analysis revealed patterns of communicating care and caring in ICU from a variety of perspectives: changing patterns of communicating care and caring, types of communication used, enablers and barriers affecting communicating care and caring, and significant issues in communicating care and caring in ICU. Figure 1 represents the patterns of communicating care and caring in ICU.

### 4.1 Changing patterns of communicating care and caring in ICU

Changing patterns in the ways that nurses communicated caring in ICU were noticeable to the researcher. First, participants indicated that changes had occurred in the way patients communicated their healthcare needs to staff. “Patients are not just receiving instructions or orders like before; patients have the right of partnership to participate and be involved in their treatment and to be informed about the progress of their condition” [P9: Interview 2].

Second, participants stressed the importance of caring communication with the patient's family by being with them, listening and responding to their needs and involving them in the decision‐making process, “It is important how you communicate with family… it is about making them feel that their care is your priority … that they [are] valued in terms of their contribution in family's conferences or meetings” [P35: Interview 3].

Third, when nurses communicated with other health professionals such as doctors, there was a sense of collegiality and equality between the doctors and nurses based on mutual respect for their levels of competency, expertise and knowledge, “Like how people talk to each other now, where you wouldn't 10 years ago. You wouldn't talk to a specialist; you'd wait ‘til you got spoken to, but now that's different. Now you can initiate the conversation with doctors” [P29: Interview].

Nevertheless, on one occasion the researcher observed a conversation between the CNM (P1) and one of the visiting doctors:

P1:
“Excuse me, can I help you? Who are you?”
Visiting doctor:
“Dr X … Why isn’t anyone following me on the ward round? On the wards, they follow me with all the pathology.”
P1:
“Well, we’re all busy and you don’t have a ward round, you come whenever you like … please don’t speak to me like that.”

P1 later spoke to the researcher with some annoyance about some visiting doctors’ behaviour in ICU:

Visiting doctors who just walk in and don’t identify themselves at all and you have the audacity to ask, “who are they.” They still think nurses are subservient but we’re not, we’re professional colleagues. You treat people with respect … it’s treating people how you like to be treated. [Field notebook 1]

Participants stressed the importance of good communication between nurses’ colleagues, irrespective of their status and role.

There is no real hierarchy … in the way staff communicate with each other…. the unit manager involves herself as a member of the team, whether she is working at the bedside or as in‐charge. Status is no barrier to the way staff communicate. [P9: Interview]

### 4.2 Types of Communication in ICU

Data analysis revealed different patterns of staff communication in ICU including verbal, non‐verbal and documentary communication.

Verbal communication was the primary means of communication in ICU. Four key aspects of verbal communication were identified: communicating with the patients, with their families, in clinical handovers and between staff members.

The researcher observed that when nurses were communicating with both patients and families, they used the “lay” language. That is, they avoided complicated technical language or medical terminology. The staff communicated in a sensitive and caring way, given the vulnerability of the patient in terms of their health and potential future. Time was offered for the patient and family to digest the information and ask questions for further clarification. In one occasion, the researcher noticed how P19 looked after her dying patient. She held the dying patient's hands and spoke to her softly:

Participant P19:
“It is ok to go, and hopefully you are not in pain. Someone is with you. you are not alone.”
Researcher:
“You are talking to her.”
Participant P19:
“I found myself privileged to care for dying patients that you spend the last few moments with. Their family members are not there and therefore, they had somebody with them.”

[P19/Researcher: Field notebook 2]

Communicating verbally with patients depended on their level of consciousness. For conscious patients, the length of interactions was significantly longer than with unconscious patients. The participants were more engaged with the conscious patient, sharing information and responding to their enquiries. With unconscious patients, the participants were observed to continue talking and explaining what was happening to the patients as they performed procedures. The focus of this interaction was primarily on treatment and management of care. Irrespective of the consciousness level of the patients, staff were caring and aware of their capacity to hear.

When the participants performed “handovers” at the bedside, the researcher noted patient involvement. Participants considered patients’ needs by giving them the opportunity to speak about how they view themselves and to ask any questions. The use of both lay and professional language was noted in these situations. Lay terminology was used when talking with patients, and medical jargon and technical language, often using acronyms for brevity, when talking to each other. This was an economical language used in an intense environment for time management. Notably, the researcher observed that the bedside handover used lay, inclusive and expansive language, with more detail of what was happening. In contrast, at the in‐charge level, handovers were economical, involved abbreviated, medical language, and used the patient's bed number or diagnosis, rather than the patient's name [Field notebooks 1 and 2].

Another caring communication was observed on different occasions, as the leaders tended to communicate indirectly to staff by sending a float nurse (is an expert RN, level two, not located to any patient, assisting the staff and in‐charge/coordinator nurse) to deliver a specific message or talk generally to staff in meetings, by using the “blanket effect,” which means not mentioning specific events or names, for two reasons. First is “to let the person involved get the message without embarrassment” [P9: Field notebook 1]. This avoids confrontation and considers the recipient's feelings. Second, this allows staff to learn vicariously from other people's experiences or mistakes. The researcher witnessed in one meeting the discussion of an incident involved the administration of a blood transfusion. Rather than naming the person/s involved, the CNM made a general statement that an incident had occurred as a result of not checking whether the blood type was the correct one for the patient. [Field notebook 2].

With non‐verbal communication, touch stood out as the central aspect of communicating caring non‐verbally in ICU. The researcher noted the importance of appropriate touch and its subsequent effects. Participants were keen to highlight the importance of assessing the context of the use of touch, which included informing the patient if touch was indicated: patients’ consciousness level, the acuity of their illness, their background, potential vulnerabilities, and the level of control and involvement in their care.

Touch can be used not only to communicate that you care, but also provides the important information about the person's physical health when undertaking a clinical assessment. “The use of touch can be a sneaky way of assessing the peripheral body temperature of the person, their heart rate…without the person actually being aware of what you are doing” [P2: Interview]. In caring for an unconscious patient, the importance of touch was raised by participants, “[With] patient who is in an unconscious state…. touch is the only way the person can sense that someone is there for them…. saying without words that you are there taking care of them” [P22: Interview].

Even when patients are sedated or unconscious, still they can feel when they are being touched. That is why we [nurses] always prepare the patient by explaining to them what we are about to do to keep them informed before touching them as part of carrying out any procedure involving touch. [P2: Interview]

When assessing and preparing the person for touch, the nurses talked about knowing whom/what/where/when to touch/ not touch. Whom to touch/when not to touch was an important consideration in the provision of care. The diversity of patients being cared for in ICU raised many issues for the staff, especially in respect of the patient's cultural/religious background, age, gender, illness severity and life experience. Therefore, participants were very cognizant that every individual respond to touch in different ways depending on their life history. One consideration is the patient's cultural background, “A person's cultural and religious background needs to be checked to identify if the use of touch is acceptable and if so, by whom” (P14). P28 spoke about the importance of assessing the appropriate use of touch:

In some faiths, touching another, especially a stranger or a person who is not a member of the family is not acceptable…. especially when the patient is female. When this occurs, the care and management of that person are entrusted to a female rather than a male staff…When a patient is a young person, there is a need to be cognisant of their sensitivities in respect of others viewing and touching their body. We are very conscious of not embarrassing them. This is also the case for elderly people, who often require care which involves touch. It is important to remember touch is meant to be therapeutic not invasive. [P28: Interview 2]

When to touch was also raised by participants as important considerations. Participants considered touch to be appropriate when patients were perceived as anxious about their situation, wanting to talk about things worrying them and looking for reassurance.

The use of touch can be a source of strength for the person in the bed that does not require conversation. It is about communicating to the patient that you are with them without having to talk. It also can be a point of affirming the person as they speak. [P8: Interview]

For patients at the EOL stage, participants discussed the importance of providing touch as a means of reassurance and comfort. “Being with patients as the person comes to that moment of death. At such times, touch has become the point of connection when words fail to express how one feels. It is in many respects a sacred moment” [P14: Interview].

Participants also pointed to using touching to build rapport and trusting relationships with patients, “By holding the patient's hand, you are trying to connect with them…building a rapport with the patient … extra bit of caring that you are more sympathetic and empathetic nurse rather than just being superficial and only professional nurse” [P17: Interview 2].

The researcher observed P15 who was looking after a patient who was fidgeting with his tube, P15 moved to the side of the bed and gently held the patient's hand and spent time talking to the patient. When questioned by the researcher on why she chose to hold his hand, she responded:

I think it is just communicating and showing someone that I am here for you and you have been heard … that you are safe …the person’s physical status can improve just knowing you are there for them, then yourself saw how the BP and HR went down. [P15: Field notebook 1]

P28 confirmed that the effects of touch extended to the patient's physical health by stating:

The use of touch has been effective in reducing patients’ blood pressure and heart rate. On many occasions, I have witnessed that the simple act of touching the patient on the hand or arm can have an immediate impact in helping them relax… and that can be seen over their faces. [P28: Interview]

Furthermore, touch is often used by staff to provide comfort and reassurance for family members, “Sometimes patients can cope with bad news more than their family members … they [relatives] need you more than the patient. By simply placing your hand around their shoulder, lets them know you are also there for them” [P34: Interview 1].

What/where to touch was important considerations raised by participants. Their foremost consideration, however, was to ensure that they did not compromise the person's integrity or self‐worth. P9 conveyed the general sentiments of the participants by stating:

The appropriateness of where to touch our patients where necessary without violating their integrity …. there are acceptable parts of the human body that touch is generally permitted and accepted…the head…shoulders, hands, legs, and feet…we need to be sensitive to how our patients feel about being touched in other aspects of their body. [P9: Field notebook 1]

The use of touch was not limited to patients and families, but also extended to colleagues in stressful time. On one occasion, P29 received a phone call that her spouse was very sick and had been admitted to hospital. Her colleagues comforted her by hugging her and asking how they could help. There was an immediate response to cover her shift so that she was able to leave work to be with her husband [Field notebook 1]. On another occasion, P31 informed the staff that her nephew and his fiancée had been killed in an accident. The staff responded with a gentle embrace, followed by an offer to be there for whatever she may have needed [Field notebook 2].

Documentary communication took multiple forms as the nurses’ notes, patient charts and a communication book. In talking with participants about the way healthcare delivery is documented, coupled with a review of what was documented, two questions were raised: “What did nurses document and why?” and “What did nurses not document and why?”

The consistent message received from participants was that any form of documentation about patient care needed to contain objective, factual and measurable data such as physical assessment outcomes, diagnostic findings, treatment interventions, management strategies, patients’ responses to treatment and the needs of the family.

Documentation… is all about recording any change in the health status of the patient and any other clinical procedures that the patient requires.…chest drains removed, and mouth, eye, pressure area care is given. It is all about recording objective facts, which is what we are taught to do. [P9: Interview 2]

**FIGURE 2:**
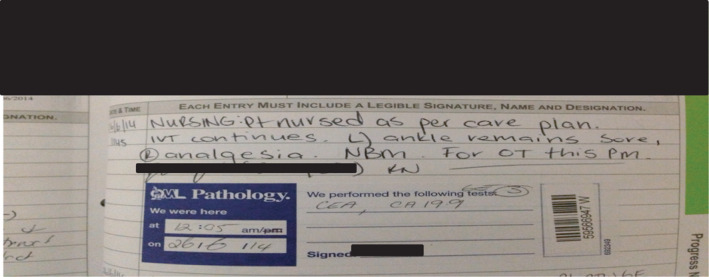
Documentation in nurses’ notes: “Patient nursed as per care plan”

The researcher noted that any reference to nursing care was either very brief or stated in general terms: “all care attended to” or “patient nursed as per care plan” [Nurses’ notes] (see Figure 2).

When questioned by the researcher about the reasons for the abbreviated manner of care documentation, P5 responded:

“All care attended to” means you have given…mouth care… eye care. However, any care involving touching the patient for the purpose of reassurance or to allay their anxiety is normally written as “psychological support given”…understood to mean that you sat down and listened to the patient and assisted the patient to feel comfortable. Such terms remove excessive documentation. [P5: Field notebook 1]

In the nursing notes, there were a noticeable language and how nurses perceived the needs of the patient.

When a patient is in need of emotional or psychological support, we would include in our documentation “patient needs a lot of tender loving care (TLC).” The use of such term would indicate to other nurses that the person is very fragile, emotional, and distressed. [P17: Interview 2]

One participant spoke of the importance of psychosocial assessment as part of unit routine, indicating, “It is very important to document psychosocial aspects of the patient's care, which is part of holistic practice… mental status, level of comfort, outcomes of visits by family” [P29: Interview].

In the Philippines, we were trained to record both objective and subjective data as part of legal requirements…there may be a misinterpretation of communication between patient and nurse… nurse and family…. leading to complaints being made… in our ICU, the way documentation works is if you do not write down what you have done, then you did not do it … not written not done.” So, I write everything down. [P14: Interview]

From the researcher's perspective, what P17, P29 and P14, indicated in the above statements, were the importance of documenting subjective data in the provision of care, as participants felt it has a place.

When questioned by the researcher about the discrepancy between what the participants described as highly valued caring practices and what they recorded, however, the participants did not all see that their caring practices were significant for documentation. Participants responded that nursing care was essentially subjugated to the realm of non‐importance in the absence of evidence, perceiving that engaging with the patient on a personal level cannot be proven to make a significant contribution to health improvement and quality of life. Although proud of their contribution to providing care, a number of participants were reticent to include such information in their documentation, as it was not considered as valuable as other information to be documented. P37 shared her thoughts about this, “Nurses don't write that they listened to the patients, touched them or held their hands… This is taken for granted as part of providing care” [P37: Interview].

I believe it is important to write down how we cared for our patients….as such information can be very helpful; however, none of the staff are going to read it, especially if it is lengthy, so we don’t write it down. It is very time‐consuming, spending too much time documenting the care instead of delivering it. [P6: Interview]

The importance of documentation in terms of legal implications was raised by participants, “Nurses are legally obligated to document every shift. Nurses’ charts and notes are legal documents…. you've got to be very careful what you document” [P13: Interview 2]. Also, several participants mentioned the importance of protecting the nurse from potential litigation, “Clear documentation can also be a protective measure in the case of treatment error or when the patient or family believe there has been a failure in care and treatment” [P32: Interview]. “We are taught to write in our charts that family have been updated about the health status of their loved one” [P2: Interview].

One particular practice noted by the researcher was the repetition in documentation, as repeating the same information in different charts. P13 also expressed her frustration at this practice in stating, “You get nurses who will document something eight times. Others and I, if we have written something somewhere, we are not going to re‐document it elsewhere because we hate to write things twice” [P13: Interview].

### 4.3 Enablers and barriers affecting communicating care and caring in ICU

**FIGURE 3:**
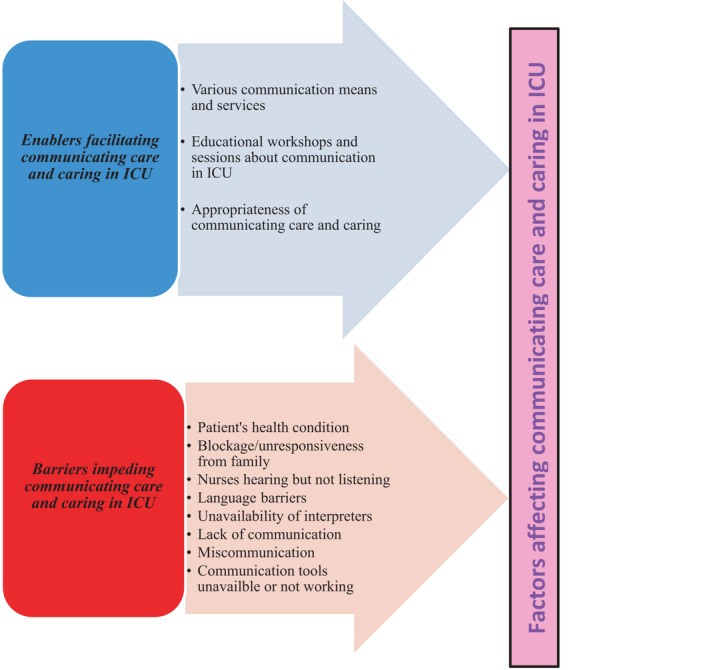
Factors affecting communicating care and caring in ICU

The data analysis revealed various factors that can affect the process of communicating caring in ICU. These factors were divided into enablers and barriers (see Figure 3).

#### 4.3.1 Enablers facilitating communicating caring in ICU

Several enablers facilitated communicating caring in this ICU. They included using a variety of communication means and services; attending unit workshops to enhance effective and caring communication with patients, their families and colleagues; and the appropriateness of communicating caring. The participants reported employing several strategies to facilitate effective communication, including the use of different tools such as mobile phones, writing boards, iPads, pictures, lip reading, leaflets and information brochures, and interpretation services when language was an issue.

Education was a major aspect of developing communication skills for staff. Participants were required to attend various workshops and educational sessions to enhance effective communication with patients and their families, including appropriate etiquette practices, especially in sensitive situations. Participants provided some illustration of the way that nurses need to communicate with their patients:

It is about looking at their [patients’] body language or eyes when they are not conscious. When they are unconscious, you need to speak clearly, keep it simple, direct to the point and usually a little bit louder because there is so much other noise going on, but not shouting at them. [P9: Interview 1]

Nurses in ICU need good communication skills …being patient…a good listener… attentive to the patient because all patients have different needs … also, the way you present yourself [in terms of] eye contact, body language, mannerisms [and] tone of voice. [P18: Interview]

Caring communication includes the importance of choosing a suitable time for nurses’ communication with their patients by respecting their unwillingness to talk—either because of being preoccupied with their illness or simply not being in the mood to engage in conversation, “Nurses need to read the patient's non‐verbal cues. Some people do not want to talk, and you must respect that…. you just have to be able to read the signs … whether your patient wants to communicate or not” [P9: Interview 1]. “We [nurses] forget that people need to rest. We need to leave them [patients] alone, for a period of time in the day… that is really an important part of healing” [P1: Interview 4].

For communication with families, P34 said, “We [nurses] need [further] education and training about how to communicate with… difficult relatives … on the psychological aspects [of caring] … about how to understand people” [P34: Interview].

Furthermore, the researcher attended several family conferences/meetings with staff members responsible for the patient issues being discussed. The doctors usually managed these meetings; however, in some cases, the expert nurse played that role—as witnessed by the researcher [Field notebooks 1 and 2].

Communication between staff was another aspect raised by participants who had experienced situations in which unprofessional and uncaring communication occurred. “Some float nurses criticise you in front of the patients when they say, ‘Hasn't anyone brushed your hair today, or haven't you had a shave?’… they just come and pick on you … that is greatly annoying” [P26: Interview].

Participants identified the importance of open communication and speaking up without fear of reprisal. At times, nurses needed to speak up by debriefing with their colleagues or talking to their CNM when there was stress or an important concern.

If you are a level 2 nurse and there is a problem…. you have a quiet chat to the nurses on the bedside … if it is like a big issue it needs to be addressed to Kerrin [CNM] who usually say: “ok … I will have a chat with them”. [P3: interview 2]

Caring communication was evident in different aspects by the provision of a suitable place for communication in an environment of respect, privacy and confidentiality. The researcher witnessed the CNM speaking individually and privately to the staff about their specific issues.

The in‐charge and float nurses’ handover is in the nurses’ station, where there are phones continually ringing and doctors coming in and out all the time…to address the situation, we said, “why don’t we give the handover in the manager’s office… where the nurses’ station is close… this will allow us privacy …to review each patient’s case”. [P5: Interview 3]

#### 4.3.2 Barriers impeding communication in the unit

There are several barriers tended to impede the ability of nurses to communicate caring for their patients. These included caring for patients who were experiencing psychosis, were unconscious, had an endotracheal tube in situ and had vision or hearing impairments. Consequently, the challenge for nurses was to create different ways to communicate caring.

When patients are confused…if a patient is trying to climb out of bed and we have to keep pushing the patient back into the bed, the patient can feel that he is not being cared for. Another thing…. when I am looking after ventilated patients, and these patients cannot communicate effectively because of their tubes. So, it’s hard to provide the emotional or physical care that they specifically need. [P24: interview]

Participants identified miscommunication as another barrier, P7 articulated:

There might be a personal clash between the nurse and patient in terms of how the nurse approaches, and the different perspectives of the situation by the patient. So, it can be purely down to miscommunication and someone's misunderstanding from either side [P7: Interview].

Participants also mentioned that sometimes communicating with the patient's family is a challenge.

Sometimes it is challenging when communicating with family … they come in and you can see that there are walls there between you [nurse] and them…Having to get through that to tell them what is going on, or to break down the wall … that is incredibly difficult and frustrating. [P3: Interview 3]

P36 stressed the importance of listening to the patient or families, as a means of communicating that they cared, “Some nurses, …. have a very busy shift and are preoccupied with carrying out procedures, which are more often than not highly technical. Turning off from what they are doing…. just we are talking about communication” [P36: Interview].

One of the significant challenges of communicating while one cares was the language barrier. When the patient does not speak English and there is no interpreter or family member available to act as an interpreter, the situation can become problematic. “Most of the time, language can be a significant barrier in communicating with patients” [P2: Interview 2]. “We have had Greek people and sometimes it was hard to get an interpreter…their family spoke English which is helpful … but their family are not here in the middle of the night and that is just a nightmare” [P37: Interview 2].

Sometimes, medical jargon needed to be used by healthcare members, which further challenged effective communication with patients and their families.

The medical staff tell patients that there are some changes in their condition, and they speak to patients in medical terms … then you see the patients just nodding their heads; then you ask them “did you really understand … do you have any questions?” As a nurse, it is your responsibility to make sure that the patient understands what is going on. [P28: Interview]

The issue of language was not confined to patients and their families but was also present for members of the healthcare team whose first language was not English.

For some nurses, their English is not easy to understand. If their accent is strong/thick or not clear or difficulties can arise….one time a doctor gave an order for insulin by phone, and it was difficult for the doctor to understand the Asian nurse’s accent …the nurse had to ask another nurse for assistance in taking the order … This was a big frustration for both the nurse and doctor. [P24: Interview]

Further, participants spoke about difficulties with communication between health professionals in ICU, whether it was verbal or written communication. “Some surgeons make your job harder; first, because they don't talk to you and second, you can't read their writing … it's illegible. So, how are we meant to know what to do?” [P36: Interview 2].

Few participants indicated other barriers in communicating caring in the handover from agency staff:

An agency staff gave me a reasonable handover, and just she was not certain about different things because she was not familiar with the paperwork and procedures of the unit. Although one of my patients was to be discharged, and I never found out until late … that my patient is going to the ward. [P27: interview]

The researcher observed some confusion in communication when working with certain groups of nurses, such as Filipino (when nodding their heads) and Indian nurses (when shaking their heads). The cultural differences between nurses of these ethnicities resulted in difficulty understanding whether they agree or not—that is, whether the head movement means “yes” or “no.” In such situations, the nurse often has to ask for verbal clarification. Additionally, some groups were sometimes observed conversing in their native language, which was neither appropriate behaviour nor appreciated by both the patient and the staff [Field notebooks 1 and 2].

Another barrier was witnessed by the researcher when the participants required the use of communication tools for engaging with patients, but the devices were found to be inoperable through flat batteries. The researcher asked three participants about the situation—none said anything about it [Field notebook 2].

### 4.4 Significant issues in communicating care and caring in ICU

The researcher observed remarkable aspects of communicating care and caring in this ICU. Seven elements concerning communicating care and caring in the unit stood out: Anticipating of undeclared individual needs for care and caring, hypocritical communication, care of the patient after death, a sense of humour, communicating care through touch, the contributions to a culture of caring by nurses from different backgrounds and a pervading sense of caring in the unit.

*The first element,* “Anticipating of the unspoken and undeclared individual needs (verbally and non‐verbally) for care and caring” is indispensable for different personnel in ICU including patients, families, nurses and other members of the health team. For instance, anticipating patient's care and caring needs is not only limited to unconscious or non‐communicative or dead ICU patients; however; it is required at all times and circumstances. Similarly, with families of patients, nurses, and other colleagues:

*For patients*, at the centre of the nurses’ professional behaviour was “The importance of reflection on practice as a means of meeting the spoken and unspoken needs of patients and their families” [P18: interview 2]. Participants P4, P18 and P15 added that “When nurses are explaining and keeping the patients well informed and meeting their spoken and unspoken needs, they are on the right path to building a trusting relationship” [P4, P18 & P15: Informal discussion/Field notebook 2]. Participants noted terms in their PAWIFs such as: “Being aware of the non‐verbal care needs of patients” [P18: PAWIF]. For example, patients’ rights were at the forefront of care as P9 stated that “Some patients do not want to talk and we as nurses must respect that; they just have to be able to read the signs and the body language—respect is all‐important.”

Almost all participants agreed that they look after their patients as they would look after themselves or a family member, “Treating others as you would like to be treated” (P1, P12, P25 & P26). P2 echoed these sentiments in stating:

In our unit, we have to ensure that patients are well looked after … the way nurses look after their patients is the way we look after ourselves and that is why we always strive to provide the best care possible. [P2: interview]

Similarly, participant P20 stated:

The nurses have considerable experience in caring for people from all walks of life and in difficult situations. Then the nurse tries to put him/herself in that situation and asks the question: “What would I like someone to do or how would I like someone to be?”. [P20: Field notebook 2]

As described by participants and witnessed by the researcher caring for patients by having a strong nurse–patient relationship, which involves being empathic, having a deep sense of being with the person during their illness and advocating on their behalf, especially when the ICU patient is unconscious and requires the nurse to anticipate what type of care is required to respond to their fluctuating healthcare needs. For example, when changing the position of an unconscious patient, it is necessary to be mindful of the importance of remaining respectful, treating the person with dignity, ensuring privacy and “Treating the person as you would like to be treated” [P26: interview 1], and “Placing yourself in the shoes of the patient” [P3: interview 1]. P37 said:

Protecting the dignity of the patient extended to family members, especially at times of treatment procedures or care interventions. With simple procedures that are not of an invasive nature or do not lead to the exposure of the patient, family are encouraged to stay. However, when this is not the case and the patient may be compromised by the family’s presence, they are generally asked to wait in the waiting room until the procedures have been completed. It is so important to protect our patients; we are their advocates at all times. [P37: interview]

P31 once viewed a nurse was performing a rough cleaning on her patient, and commented:

People do not think a lot when they do simple things like when they wipe a patient’s bottom, some nurses are really rough. Would you wipe your own bottom that hard? They should consider what it is like being in the patient’s position. [P31: interview]

A number of participants identified being a patient advocate as one of the fundamental characteristics of the ICU nurse. As patients in ICU are vulnerable and usually unconscious or sedated, nurses must act on their behalf, “Usually ICU patients are critically ill and unconscious … they can't look after themselves … their lives are in our hands, so we are their ears, eyes, hearts and advocate” (P2), “Being the patients’ ears, eyes and heart and acting on their behalf” (P7 & P12).

Several participants spoke of the tension between team members about EOL choices for patients. The nurses interviewed considered prolonging life as an unnecessary trauma to the patient and their family, while the medical staff viewed maintaining a person on life support as sustaining life. P10 expressed her view:

At times I get annoyed at what happens in the unit in terms of keeping people on ventilators. I do not like how doctors push the patients to live longer. I feel sometimes we are not doing them any justice to keep them going by prolonging the inevitable. I feel sometimes it is a bit cruel. I can understand why we do it because the family does not want to let their loved one go yet, and it is nice for the family to say goodbye. I think it becomes a bit selfish of the family to let the patient suffer. Ultimately, caring is making sure your patient has the opportunity to have the best outcome in their current situation. [P10: interview 4]

At times, nurses perceived doctors to be prolonging the life of a patient at the behest of the family when the prognosis was terminal. Participants spoke of the difficulty they experienced at having to be part of this because the family were not prepared to face the reality that their loved one was about to die. In such situations, participants felt torn between providing quality EOL care. Sentiments were expressed by P10:

We [nurses] do not like when a decision is being made by doctors not to do anything for the patient, except for prolonging the inevitable, simply for the sake of the family who is not prepared to let them die. We appreciate that the family has their needs, but it should not be at the expense of the patient, especially when you can see the patient no longer has any quality of life. We [nurses] are all on one page in this regard but our hands are tied. It is the decision of [the] medical treating team in consultation with family. Such decisions are not easy when there is a difference of opinion. [P10: interview]

*For family*, at the centre of the nurses’ professional behaviour was “the importance of reflection on practice as a means of meeting the spoken and unspoken needs of patients and their families” [P18: interview 2]. Participants pointed out to the undeclared needs of the family by involving them in providing care to their loved one as a means of letting the relatives be close to them. Give them the opportunity to feel that they have been of assistance and helpful is better than leaving them feeling impotent and unable to help, “We used to involve the family in the patient's care, because sometimes they just feel so helpless, so if they can do even a tiny little thing it relieves them” [P12: Interview]. However, most staff were attentive to the unspoken needs of the family. On one occasion, the researcher observed P15 was bringing a chair to the patient's wife and assisting her to take a seat. P15 lowered the patient's bed to the same level as the seat of the relative. Participant P15 spoke to the wife in a very kind and warm way and asked if she would like a cup of tea, while putting her hand on her shoulder. P15 payed attention to the relative's needs and comfort, which is an indirect discomfort for the patient too [P15: Field notebook 2].

*For colleagues*, there was an underlying atmosphere of assistance and collegiality and the nurses supported each other both physically and emotionally. In particular, when caring for difficult patients or family members can add a heavy workload for the nurse. P29 said:

Most of the time we look after each other when someone’s tired, upset, stressed, or needs a bit of help… We keep a bit of an ear out if someone’s getting a difficult patient. To make sure you go and give them assistance…or if they’re busy, you go and see if they want a drink or a cup of tea, because sometimes it’s a bit hard to get away. [P29: interview]

Participant P7 shared her thoughts in commenting on the importance of communicating the needed respect between staff, “Respect is at the heart of caring for others, but respect needs to be communicated to others in the way you interact and work alongside other members of the team” [P7: interview].

On other occasions, participants expressed their caring for doctors in ICU, P16 stated: “We look after our doctors who work 24‐hr hard shifts.” P3 captured how they care about their doctor colleagues when on‐call:

The in‐charge looks around and gathers all the information and try to sort things as planned, but if that doesn’t work and it needed to be attended, then the in‐charge goes around the whole unit and says: “has anyone got anything for the on‐call doctor?” and then [they] cluster it all together and do it at one time, rather than calling him every 45 minutes about something. [P3: interview 2]

Briefly, nurses need to put themselves in the positions of patients, families and colleagues to understand their needs by asking: "If you are a patient/family/colleague, what do I need?"

*The second element,* the “hypocritical communication,” means showing the opposite of what is hidden. For example, nurses show others positive communication in care and caring that runs counter to their internal feelings. The researcher witnessed the “hypocritical communication” in few scenarios. *Firstly*, P29 was allocated to a patient who was a former nurse. This patient was admitted to ICU following a drug overdose because of her addiction. The researcher observed P29 treating the patient as an inferior and relaying the patient's story to her colleagues in unprofessional manner. As both a researcher and a nurse, the researcher felt so upset about the participant's mannerism, which was totally the opposite of what P29 articulated that she “cares for the patient as she was in his /her position” [P29: Field notebook 1]. In that situation, the researcher needed to control herself at that point and chose to ask the participant about her attitude in the interview. When asked about this incident, the participant's explanation was accusatory of the patient being addicted. The researcher found it difficult to not respond, as she wanted to focus on listening to the participant's side of the story [P29: Field notebook 1]. *Secondly*, during one period of observation, three staff were attending to one patient, who was experiencing some faecal discharge and required a complete linen change. Between ensuring that the endotracheal tube (ETT) and monitor leads remained in place, the staff were able to carefully negotiate cleaning up the patient and changing the bed linen, although the nurses seemed to be experiencing some discomfort at the odour. However, despite their dislike for these tasks, staff did not allow their personal aversions to interfere with the quality of care provided and they did not let the patient become aware of how they felt. P22 was overheard speaking to a patient who had been rolled onto one side, away from view of the nurse's facial expressions. P22 spoke to the patient in a sensitive manner, asking him, “do you want to defecate?” The patient replied “yes,” and P22 responded, “OK. One moment; I will get you the bed pan.” However, the nurse's facial expression and body language indicated that this was not a likeable task. [P22: Field notebook 1]. *Thirdly*, during a bronchoscopy procedure in ICU, the surgeon and P11 discovered that a tiny piece of the bronchoscope was missing, and they needed to get another bronchoscope. As an observer with experience in the operating theatre (OT), the researcher suggested use of a three‐way stopcock connection to address this problem. The surgeon and the nursing team appreciated this idea at that time and obtained the three‐way stopcock, which rectified the problem. Unfortunately, it was later discovered that P11 complained about the intervention to the CNM. This incident made the researcher very careful about her participation, even when it was useful. She reminded herself to remain in her role as a complete observer and informed the CNM that she would not interfere in the future. This incident affected the researcher for several days, and she reflected in the field notes to continuously remind herself to be cautious in her research role while conducting this study.

*The third element* means showing the manner in which a person who had died was cared for posthumously. The researcher witnessed this on several occasions, noting that the staff attending to the final preparation of the patient were extremely respectful of the person before them. During corporal preparation of the deceased, which required dismantling and removal of the technology surrounding them, the nurses continued to honour the body as if the person was still alive. Words of comfort and support were replaced with a respective attention to preparing the body for transportation to the mortuary [Field notebooks 1 & 2].

*The fourth element,* a sense of humour, was evident during the observations to neutralize the daily stresses and difficult times faced by the staff. Whether in the form of joking, making what they perceived to be clever comments in the form of retorts, or responding to comments of staff that elicited a smile, humour served as an antidote for coping with life and death situations. It also assisted staff in maintaining a sense of confidence, whether that be with the patient, family or staff. P37 captured the general participants’ feelings concerning the humour:

Humour and laughing make all of us feel at ease. Whether the patient, family or staff, humour plays an important role in making a dark place more light‐filled. It helps us get through the day, which is often filled with difficult events and decisions. Humour often replaces feelings of not being confident with feeling confident. [P37: Interview 3]

*The fifth element*, amid what initially appeared to the researcher to be a culture of “standoffishness” (e.g. being more cerebral than emotive, more technical than person‐centred, and with a focus on task completion rather than holistic care), became increasingly apparent that this was not the case. In essence, the unit was a place where touching formed the conduit of care and caring, as previously discussed.

*The sixth element,* nurses varied background and training all contributed to a culture of caring in this unit, is something that the researcher had come to expect after many years working in ICUs in different countries. Each person brings their own style of caring, some care more and some care less. In this instance, nurses who were trained in England often were observed as providing quality care that exceeded that provided by other team members. One of the characteristics of this particular group that stood out for the researcher was their ability to communicate and connect with patients and their families through what the researcher observed as a “quiet presence”: talking to them in quiet and respectful tones that seemingly invited both patient and family to share their concerns and hopes for recovery.

*The seventh element*, apart from each of these elements, was a pervasive sense experienced by the researcher, which, for her, defied description. It was as if each of the elements converged to make an unspoken statement that this unit valued caring communication as an essential mechanism in creating a culture of caring in the unit.

## 5 DISCUSSION

The participants’ perceptions and experiences of communicating the care and caring in ICU are represented in these findings: changing patterns and types of communication used with a diversity of people in ICU, various enablers and barriers affecting communication in ICU, and aspects of caring communication that were particularly visible to the researcher.

In the current study (CS), the researcher identified different patterns of communicating caring between nurses, nurse–patients/families, nurse–unit managers and nurse colleagues. In a hermeneutic study, Karlsson, Forsberg, et al. (2012) explored the ways in which nurses communicate with MV patients in ICU and evaluated whether such communication is considered caring. The findings of their study revealed several ways nurses communicate that they care for their patients, including being attentive and watchful, taking note of the patient's non‐verbal communication, being inclusive, using humour to introduce moments of light‐heartedness in stressful times and creating a sense of security for the patient by the quality of care provided. These findings were congruent with the CS findings.

The findings of the CS showed nurses employed a range of strategies to communicate with MV patients who were unable to communicate verbally as several devices were made available to those patients to assist them in communication. Also, partial or complete tube cuff deflation with digital occlusion, plugging or capping of the tube, a one‐way speaking valve and tracheostomy button were used. In addition, other measures were found in other studies (Flinterud & Andershed, 2015; Morris et al., 2015; Pina et al., 2020; Shiber et al., 2016; Ull et al., 2020). The use of such devices, however, is predicated on the ability of the patient to be able to use them. In the CS, MV patients and who also had other injuries as injured or oedematous hands were unable to use hand‐held devices. Then, nurses were required to use non‐verbal communication such as direct eye contact, nodding and lip reading. These are consistent with the findings of Karlsson, Forsberg, et al. (2012) that irrespective of the health status and communication limitations of the patients, nurses spoke of the importance of being able to “be there” for their patients, working to find the most convenient and appropriate way to communicate and anticipate patients’ needs. Interestingly, the CS findings on “anticipating the undeclared personal (patients, families, colleagues) needs for care and caring” was proclaimed earlier by Leininger (1991, p. 4), who defined caring as “those actions and activities directed toward assisting, supporting, or enabling another individual or group with evident or *anticipated* needs to ameliorate or improve a human condition or lifeway.”

In the quantitative study of Saldaña et al. (2015), the scholars discovered that communication between ICU nurses and patients highlighted the importance of intra/inter/transpersonal communication, in which communication is made with comprehension, empathy, acceptance, authenticity and respect to establish a therapeutic relationship that identifies, comprehends and satisfies patients’ and their families’ psychosocial needs. These findings align with the CS findings, in which participants shared a cultural view of the importance of being empathic, respectful and authentic in all communications with patients and their families.

For the communication among staff in ICU, apart from documentation, the most common devices used to enable rapid updates of patient information and distribution to all members of staff were smartphones and pagers. Staff were frequently observed checking their work emails, smartphones and pagers for information updates, showing a new emerging culture of communication. Similar findings were evident in other studies (Al‐Qadheeb et al., 2013; Curry, 2012).

In the CS, barriers to communication were related to the patients’ health status, level of consciousness, mechanical ventilation, lack of access to communication aids and either being too weak or depressed to attempt to communicate. Participants spoke of those difficulties facing both the patient and staff in providing the right care in a timely and respectful manner. This is consistent with the findings of previous studies (Happ et al., 2011; Magnus & Turkington, 2006). Several studies discussed the challenges communicating with ventilated patients. Alasad and Ahmad (2005) used a phenomenological design to investigate Jordanian critical care nurses’ (CCNs) verbal communication with critically ill patients. Data were gathered through interviews and participant observations. The findings revealed that nurses tended to use less verbal communication when caring for unconscious patients than they did with verbally responsive patients. Nurses also communicated less with unconscious patients than they did with conscious patients, which contrasts to the findings of the CS, in which participants stressed the importance of verbally communicating with the unconscious patient when carrying out nursing procedures as a point of respect for the person, even in the patients’ EOL stage. The variance of the findings between the CS and Alasad and Ahmad (2005) investigation could be due to the date of the latter study or cultural differences.

A quantitative study was undertaken by Happ et al. (2011) to describe the communication interactions, methods and assistive techniques between nurses and non‐speaking critically ill patients in ICU. These researchers used video recordings and patient self‐rated ease of communication questionnaires. They found that communication difficulty was the greatest stress for MV patients and nurses, which is consistent with the CS findings.

A further barrier to communication in the CS was staff handovers, in which being unable to understand handover notes written in medical jargon, which was also described as a major impediment to receiving a comprehensive handover of the patient's health status and treatment regime, especially for new or agency staff. Similarly, the difficulty in understanding handover notes between nurses and others was also identified in a multicentre pilot study by Magnus and Turkington (2006). These researchers investigated patients’ and ICU staff's (e.g. doctors, nurses and allied health professionals) perceptions and experiences of communication interactions in ICU.

A language gap has also been identified as a barrier to caring communication. A phenomenological study undertaken by Coleman and Angosta (2017) related to CCNs caring for patients and families with limited English proficiency. In such situations, the use of interpreters—either in‐person or via telephone—was considered preferable to struggling to understand the need of patients to provide appropriate care. These findings are consistent with those of the CS, in which interpreters were used when and where available. When interpreters were not available, the staff had to rely on family or friends to interpret the patient's needs.

In the CS, the identification of the need for communication training programmes for ICU staff, especially for communicating with non‐verbal patients, is consistent with the findings of other studies (Magnus & Turkington, 2006; Shiber et al., 2016).

In a qualitative analysis of healthcare professionals’ perspectives on communicating with patients’ families in ICUs, Schubart et al. (2015) identified potential barriers to facilitating caring. Their findings are consistent with those of the CS, in which miscommunication between members of the treating team and patients, limited time, work overload and language were viewed by participants as impeding the provision of quality care.

Interruptive communication is a significant cause of mistakes and can have consequences that affect the provision of patient care in ICUs (Alvarez & Coiera, 2005, 2006; Manojlovich & DeCicco, 2007). For example, the findings of two Australian studies by Alvarez and Coiera (2005, 2006) revealed that communication interruptions could affect retention of important information about patient health and treatment and thereby place patients at risk. Alvarez and Coiera (2005) observations and conversations with ICU nurses and doctors indicated that distractions during handover were likely to lead to miscommunication. Similarly, participants in the CS spoke of the disruptive nature of handover and described how such disruptions interfered with receiving an accurate report about the health status and management of patients.

Loghmani et al. (2014) explored the factors that influence communication between nurses and families in ICU. The findings of their study identified barriers to communication between nurses and the patient's family, including misunderstandings about treatment needs, and conflicts between patients’ family members about treatment options. These findings are consistent with those of the CS, in which participants struggled to communicate with some difficult families.

Reid et al. (2011) investigated communicating the news of a patient's deaths to families. They stressed the importance of the presence of a nurse at that time to support the families and doctors. Nurses were perceived as more available than doctors in critical care settings; thus, they were in many respects a conduit between the doctor and the patient's family when clarification of information was required and to provide support at such a critical time. Nurses were also well‐positioned to facilitate the family in saying their farewells by encouraging families to touch, hold or kiss their deceased loved one. Similar in the CS, several participants shared their experiences of providing family support prior and, after their loved one had died, even to the point of attending the patient's funeral as observed by the researcher.

Participants in the CS identified numerous communication enablers such as the importance of open communication and speaking up. This is congruent with the findings of Reader et al. (2007), who conducted a cross‐sectional survey of ICU nurses and doctors, in which communication openness was associated with the degree to which staff understood patients’ care goals.

For documentation, the researcher found the participants documented vital signs, haemodynamic parameters, diagnostic tests, and procedures by physicians, nursing activities and interventions. This is consistent with Bloomer and O'Connor (2012) findings that ICU nurses document haemodynamic parameters and clinical interventions.

Carelock and Innerarity (2001) suggest that complete, clear, accurate and concise records of nursing care are a powerful tool in assuring quality patient care in ICU, which is consistent with the articulations of participants in the CS, but not evident in their documentation of patient care.

In the CS, participants used non‐standard abbreviations and repetitious information, which is congruent with the findings of Paans et al. (2010), who used a cross‐sectional retrospective patient record review to measure the accuracy of nursing documentation in 35 wards from seven different hospital specialty areas (e.g. two ICUs) in the Netherlands. These authors found that more than 50% of evaluations contained unnecessary wording and non‐standard abbreviations that could be misinterpreted. Paans et al. (2010) suggested that nursing documentation should be understandable and presented in a logical order and that reports involving repetitious, redundant content were time‐consuming to read. Additionally, using abbreviations and acronyms in documentation is problematic, as it might lead to misinterpretation and mistakes in medical records (Beach & Oates, 2014; Dimond, 2005).

The absence or lack of documentation of nursing care was noticeable to the researcher and has been described in other studies. For example, Weyant et al. (2017) found that nurses rarely document their actual nursing care of patients (e.g. touch and its impact on patients). When evaluating nursing documentation of patient hygienic care, Inan and Dinç (2013) found the consistency between the actual care given by CCNs and what was documented in nursing records and the consistency was 77.6%, and documentation was poor and incomplete. In another descriptive study by Borsato et al. (2011) based on secondary data of nursing quality assurance at an institution from 2002–2009, the researchers found that although ICU nurses’ notes were used to record information on care delivery, generate communication among the health professionals, allow the continuity of their work process, guarantee security to patients and facilitate support from legal and ethical viewpoints (Borsato et al., 2011), it was still inadequate or incomplete and did not meet the ICUs’ nursing documentation criteria. Similarly, in a retrospective record review by Goss et al. (2011) of documented oral care practices in an ICU, the researchers identified a lack of detailed oral care documentation in patients’ medical records. Further, in a review of charts of adult ICU patients by Kirchhoff et al. (2004), comprehensive documentation of EOL care was found to be lacking. The findings of these studies are consistent with those of the CS, in which nurses’ documentation in terms of detailed information of nursing care was often either absent, abbreviated or incomplete.

Nurses not documenting caring activities in patient care records was rationalized by participants in the CS. They suggested that subjective information about the way they care for patients is not valued by the healthcare team; therefore, providing care was prioritized over documentation of care. These findings are similar to those of Gugerty et al. (2007), who found that nurses prioritized patient care over documentation of subjective data that they considered unnecessary.

Participants in the CS stressed the importance of documentation for different reasons. One was to avoid claims of negligence by recording important data that needed to be communicated to other health professionals. As Carelock and Innerarity (2001) noted, documentation is essential for nurses as advocates for their patients. Failure to document effectively could result in unsafe practice and litigation. Furthermore, the importance of documenting care can be summed up in two aphorisms: “If it's not written down, it didn't happen” (Andrews & St Aubyn, 2015, p. 22) and “If it wasn't documented, it wasn't done” (Frank‐Stromborg et al., 2001, p. 842). These findings are congruent with those of the CS as “not written not done.”

One question remains from the researcher's perspective: if caring is considered fundamental to nursing and is a valued practice in ICU culture, why are caring behaviours undocumented? The nurses do not have a model for documenting caring behaviours in ICU, which suggests a need to consider development of standard documentation charts for nurses’ caring behaviours in ICUs.

### 5.1 Limitations

The only limitation for this study is that the findings could not be considered generalizable to the broader population because of the cultural differences in organizations and critical care settings. However, this was the expectation of the study design.

## 6 CONCLUSION

To my knowledge, this is the first study to examine communicating care and caring in ICU inclusively, including exploring the patterns of communication, changing patterns of communication, various patterns of communication used, enablers and barriers of communication and significant issues in communicating care and caring in ICU such as “anticipating of undeclared individual needs for care and caring,” “hypocritical communication,” “care of the patient after death,” “sense of humour,” “communicating care through touch,” the “contributions to a culture of caring by nurses from different backgrounds” and “pervading sense of caring within the unit.” Moreover, documentation of the patients’ psychological and emotional needs and nurses’ responses needs further consideration. Furthermore, this study addressed communicating care and caring widely to include patients, families, nurses and other health professionals in ICU. These findings require a determined effort and consideration from all stakeholders, including nurses, clinicians, educators, researchers, managers and policymakers.

## CONFLICT OF INTEREST

The author declares no potential conflict of interests to the research, authorship or publication of this article.

## Supporting information

File S1

## Data Availability

​The data that support the findings of this study are openly available in Wiley Online Library at http://doi.org/10.1002/nop2.1061, reference number [NOP21061].
